# Recent Developments in the Search for Urinary Biomarkers in Bladder Cancer

**DOI:** 10.1007/s11934-017-0748-x

**Published:** 2017-11-13

**Authors:** Aaron Leiblich

**Affiliations:** 1 Nuffield Department of Surgical Sciences, John Radcliffe Hospital, University of Oxford, Headington, Oxford, UK; 2 Department of Urology, Churchill Hospital, Oxford University Hospitals NHS Foundation Trust, Headington, Oxford, UK

**Keywords:** Bladder cancer, Urinary biomarkers, Extracellular vesicles, Exosomes, MicroRNA

## Abstract

**Purpose of Review:**

This review aims to evaluate research surrounding the utility of urinary biomarkers to detect bladder cancer and predict recurrence.

**Recent Findings:**

Recent research has focussed on the evaluation of genetic markers found in urine to provide diagnostic and prognostic information. Furthermore, the isolation and characterisation of extracellular vesicles (EVs) from the urine patients with bladder cancer provide an exciting new development in biomarker research that is set to expand in the coming years.

**Summary:**

Current urinary biomarker research is a broad field that encompasses the evaluation of urinary proteins, DNA, RNA and EVs to detect signatures that can be used to predict the presence of bladder cancer and provide prognostic information. EVs in particular offer an exciting and novel perspective in the search for accurate bladder cancer biomarkers.

## Introduction

Bladder cancer is the 7th commonest cancer worldwide to affect men and the 14th commonest cancer to affect women. Rates of bladder cancer appear to be higher in the developed world, where it represents the 4th commonest cancer in men [1]. The UK has the lowest incidence of bladder cancer in men in Europe and the 14th lowest for women [2]. Bladder cancer accounts for 3% of all cancer deaths in the UK, with a crude mortality rate of 8.2 cancer deaths per 100,00 individuals [3].

The presenting feature of the majority of new diagnoses is haematuria, and diagnostic workup for haematuria includes cystoscopy and upper tract imaging to detect urinary tract malignancies. Furthermore, cystoscopy is utilised in the follow-up of patients treated with transurethral resection of bladder tumour (TURBT) for non-muscle invasive bladder cancer (NMIBC) as part of a surveillance strategy to detect disease recurrence. Overall, cystoscopy is an effective tool for detecting bladder cancers and is a procedure associated with low morbidity. However, infection rates after flexible cystoscopy is in the region of 3% [4], whilst dysuria (50%), frequency (37%) and visible haematuria (19%) are experienced relatively frequently after flexible cystoscopy [5].

Although cystoscopy remains the gold standard for detecting bladder cancers, effectiveness is operator dependent and sensitivity and specificity of white light cystoscopy range from 62 to 84% and 43–98%, respectively [6]. Indeed, the detection of small papillary tumours, satellite lesions and CIS is known to be suboptimal with white light cystoscopy, both at initial diagnosis and during surveillance for recurrence [6]. There has been a move in recent years to develop adjuncts to white light cystoscopy to improve diagnostic accuracy and detection of recurrence, including the emergence of photodynamic diagnostic (PDD) cystoscopy [6] and narrow band cystoscopy [7]. In a similar vein, the field of bladder cancer biomarker research is focussed on developing non-invasive and cost-effective strategies that can be employed to aid and improve detection of bladder tumours. In particular, urinary biomarkers may yet prove particularly useful diagnostic adjuncts in bladder cancer as urine-based diagnostic tests offer a non-invasive and potentially cost-effective method of improving bladder cancer detection rates.

The object of this article is to familiarise the reader with the current landscape of research in the field of bladder cancer urinary biomarkers. I will first focus on describing rather more established biomarker tests and their effectiveness in detecting bladder cancer. I will then draw attention to important and new avenues of biomarker research that have emerged in the literature in recent years, with a view to speculating how this research strand may develop over the next few years.

## Urinary Biomarkers for Bladder Cancer—Where Are We Up to?

For several decades, an important aspect of bladder cancer research has been focussed on the search for urinary biomarkers that can be utilised to detect bladder cancer with a high degree of specificity and sensitivity, in the hope of developing non-invasive diagnostic tests that can be employed to aid the detection of new tumours and also predict recurrence after treatment. The current gold-standard investigation for detecting bladder malignancies is cystoscopy, a procedure that is invasive and may induce anxiety and cause discomfort in patients undergoing the test [8]. Clearly, a non-invasive test that performs as well as cystoscopy would be a very attractive prospect.

Below, I shall provide an overview of biomarker tests that have already been established and scrutinise the effectiveness of these techniques for detecting bladder cancer. This discussion is by no means exhaustive in terms of the variety of urinary biomarkers that exist, but it does include an analysis of the most prominent and widely utilised non-invasive urinary tests for bladder cancer.

## Urine Cytology

Urine cytology still remains the most accurate non-invasive test for bladder cancer routinely used in clinical practice. The sensitivity of urine cytology for detecting urothelial cancer ranges from 25 to 95% [9–14]. Indeed, urine cytology is effective for the detection of high-grade and high-stage disease. Urine cytology displays a sensitivity and specificity for high-grade lesions and carcinoma in situ (CIS) of 80–90% and 98–100%, respectively [10]. However, urine cytology proves relatively ineffective as a tool to detect low-grade malignancy. One review calculates that cytology has a sensitivity and specificity for detecting low-grade disease of just 0–100% and 6–100%, respectively [15]. Furthermore, benign conditions that elicit inflammatory changes within the bladder can induce changes in cellular morphology that are difficult to differentiate from cancer by urine cytological analysis, resulting in a false-positive rate of up to 12% [16].

## Fluorescence In Situ Hybridisation as a Probe for Bladder Cancer

In 2000, a novel fluorescence in situ hybridisation (FISH) probe set was developed for the detection of bladder cancer, referred to as UroVysion [17]. This technique involves the analysis of exfoliated urothelial cells present in the urine and examines them for aneuploidy of chromosomes 3, 7 and 17 and loss of the 9p21 locus, all of which chromosomal aberrations commonly observed in bladder cancer cells. A large meta-analysis demonstrates that UroVysion possesses a sensitivity and specificity of 72 and 83%, respectively, for the detection of bladder cancer. Furthermore, a positive UroVysion test after the completion of BCG therapy is associated with treatment failure. Indeed, a positive test at the end of BCG treatment in patients with superficial disease denotes a higher risk of progression to muscle invasive cancer [18–20]. Overall, FISH displays a relatively high specificity for detecting bladder cancer and appears that it may have some use for predicting recurrence. However, the sensitivity for picking up low-grade cancers remains relatively low at approximately 41% [21].

## Urinary Immunoassays for the Detection of Bladder Cancer

An adjunct to urine cytological analysis, known as ImmunoCyt/uCyt+, was developed in the late 1990s in an effort to improve non-invasive detection of low-grade disease. This technique employs fluorescent-labelled antibodies that are directed against three antigens that are commonly expressed by exfoliated malignant urothelial cells. These antigens include a glycosylated form of carcinoembryonic antigen (CEA) and two mucins [22]. Whilst the sensitivity of cytology for detection of urothelial malignancy may be boosted from 50 to 90% by employing ImmunoCyt/uCyt+, the specificity is less than that achieved by employing cytology alone (72 vs 80%) [23].

An immunoassay based on the detection of nuclear matrix protein 22 (NMP22) in urine was developed in the USA in the mid 1990s. NMP22, a marker of urothelial cell death, is frequently elevated in the urine of patients with bladder cancer. This test possesses a sensitivity of 51–85% and specificity of 77–96% for the detection of bladder malignancies [24, 25]. However, false-positive results may be encountered in the presence of haematuria and benign inflammatory conditions affecting the bladder [26, 27].

## Additional Protein Markers Detectable in Urine

In addition to the immunoassays discussed above, a whole host of proteins shed into urine by cancer cells have been characterised, but most have proven rather variable in their effectiveness to detect cancer with any consistency [28]. However, there are a selected number of urinary protein markers that appear to buck this trend and may possess some robust diagnostic value. For example, several immunological assays have been developed to detect the presence of fragments of cytokeratin 8 and 18 in the urine [29, 30]. The cytokeratins are intracellular proteins that form part of the cytoskeleton of epithelial cells. Urothelial cytokeratins are released to into the urine after cell death and as such can predict the presence of cancer. Testing kits utilising antibodies against these cytokeratins display a sensitivity ranging from 50 to 61% and a specificity ranging from 63 to 97% [30]. As with many of the strategies already discussed, immunoassays based on cytokeratin detection suffer from relatively high false-positive rates and limited ability to detect low-grade tumours [31].

Two transcription factors, BLCA-1 and BLCA-4, demonstrate elevated expression early in the development of bladder cancer and show some promise as potential biomarkers. In particular, BLCA-4 appears to be selectively expressed in the urothelium of bladders containing tumours (both in cancer cells and adjacent benign urothelial cells) but not in bladders unaffected by cancer. ELISA assays detecting the presence of BLCA-4 in urine have a sensitivity of 89–96% and a specificity of 90–100% [32–36], whilst an assay for urinary BLCA-1 demonstrates a sensitivity of 80% and specificity of 87% [32]. Although these markers appear to show some promise as an adjunct to diagnosing early tumours, further validation is required [37].

## The Use of Genetic Material as Biomarkers for Bladder Cancer

An array of genetic material derived from urothelial cells can be detected in urine, including DNA, RNA and micro-RNAs. In recent years, interest has arisen in the idea that detection of signatures in genetic material isolated from urine could help predict the presence of cancer and/or monitor response to treatment.

Although bladder cancers display a great deal of genetic heterogeneity in comparison to many other types of tumour [38], non-muscle invasive bladder tumours display a high frequency of mutations in the *FGFR3* oncogene, resulting in dysregulation of the RAS-MAPK pathway [39, 40], whilst mutations in the *RAS* oncogenes (*HRAS*, *KRAS*, *NRAS*) are observed in approximately 13% of all bladder tumours [41, 42]. Indeed, *FGFR3* mutation analysis of voided urine has been performed and demonstrated a sensitivity of 58% for detecting bladder cancer. Whilst this does not compare favourably to some of the immunoassays described earlier, it is worthwhile noting that a positive test result was associated with nearly a fourfold increase risk of recurrence. Therefore, *FGFR3* mutation analysis may hold some promise as a non-invasive tool for diagnosing and predicting recurrence [43, 44].

## Detection of Epigenetic Alterations in Urine as a Diagnostic Tool in Bladder Cancer

Epigenetic alterations are a frequent observation in most cancers [45] and the most well-characterised epigenetic phenomenon is DNA methylation. Alterations in DNA methylation are often observed in cancers and may lead to dysregulation of gene expression. A broad array of disparate genes has been identified in bladder cancers that display elevated levels of methylation in their promoter regions [46–48]. Although the role that methylation of these genes play in bladder cancer has yet to be characterised, there is some interest in whether the methylation status of these genes can be utilised as a diagnostic signature for detecting cancer.

A small number of studies have sought to detect methylation markers in the urine of patients with bladder cancer. For example, one study found that detection of hyper-methylation of the genes VAX1, KCNV1, TAL1, PPOX1 and CFTR in DNA found in urine was able to predict primary and recurrent disease with a sensitivity of 89% and a specificity of 88% [49]. Although the specificity of methylation markers is very encouraging, the molecular genetic techniques to detect epigenetic alterations are still rather expensive, time consuming and highly specialised, meaning that testing for methylation markers as a routine clinical test is not currently practical.

## Urine-Isolated RNA as a Biomarker

A nascent field in urinary biomarker research is the detection of urothelia-derived RNA in voided urine. Interestingly, detection of surviving (an anti-apoptotic protein) mRNA in urine has reasonable sensitivity (64–83%) and specificity (88–93%) for detecting bladder cancer, although, rather like many of the protein-based assays, sensitivity for detecting low-grade disease is relatively limited [50–52]. Micro-RNAs (miRNAs) are non-coding RNA sequences that are involved in regulation of gene expression by modulating the activity of mRNA molecules by targeting them for degradation. Profiling of a panel of 6 miRNAs detected in urine has been performed and shown to accurately diagnose bladder cancer with a sensitivity of 83% and specificity of 87%. Research into the utility of miRNAs as biomarkers of disease is still at an early stage and more research needs to be conducted to define accurate miRNA signatures.

## Recent Advances in Urinary Biomarker Research

The last year has seen interests emerge in the role that cancer cell-derived extra-cellular vesicles (EVs) play in disease progression and micro-environmental remodelling. EVs are commonly classified based on size and include micro-vesicles, oncosomes and exosomes. There has been considerable interest in recent years in the role that exosomes in particular play in cancer development and progression. Exosomes are membrane-bound nanoparticles secreted by most cell types and contain cargoes that include genetic material (e.g. DNA, mRNA and miRNA) and proteins. As such, exosomes appear to function as vehicles that can effect inter-cellular signals and also transfer genetic material from one cell to another. Indeed, many cancer types have been shown to secrete increased levels of exosomes and cancer-derived exosomes have been implicated in induction of angiogenesis, extra-cellular matrix remodelling and ‘priming’ of distant pre-metastatic niches [53].

There have been encouraging reports in the last couple of years of groups successfully isolating EVs from human urine detecting differences between the profiles of EVs in patients with bladder cancer versus healthy controls. For example, an interesting micro-fluidic chip-based system has been employed by one set of researchers to isolate and analyse EVs from patients with and without bladder cancer. They demonstrated that the concentration of EVs in urine from patients with bladder cancer was significantly higher compared to healthy controls. Indeed, this technique displays a sensitivity of 81% and specificity of 90% for accurately detecting bladder cancer [54].

In addition to studying alterations in the concentration of EVs found in the urine of patients with bladder cancer compared to healthy controls, an alternative research strategy seeks to characterise the cargoes contained within EVs and determine whether specific profiles can be predictive of cancer. One such approach has been to determine whether EVs isolated from bladder cancer cells contain unique proteins that can be used to differentiate them from EVs isolated from healthy tissue. Indeed, proteomic approaches have successfully characterised proteins carried within or on the surface of EVs isolated from bladder cancer cell lines and from urine of patients with bladder cancer [55]. One proteomic analysis of urinary EVs identified 2 proteins, alpha-1-anti-trypsin and H2B1K, which are enriched in EVs isolated from patients with bladder cancer [56]. Additionally, H2B1K was shown to have some prognostic value as 3-fold elevations in the levels of this protein accurately predicted recurrence.

One recent study sought to isolate genetic material, specifically long-non-coding RNAs (lncRNAs), from urinary exosomes [57]. They showed that the levels of lncRNAs in urinary exosomes were elevated in patients with urothelial cancer compared to healthy controls. One lncRNA of particular interest is the tumour-associated lncRNA HOTAIR, which the authors show that it plays a role in mediating the epithelial-to-mesenchymal transition (EMT), a process that heralds a phenotype typified by increased cancer cell invasiveness and migratory behaviour.

Another recent study sought to analyse the specific composition of miRNAs and proteins associated with urinary EVs in patients with bladder cancer. Urinary EVs were isolated from both healthy individuals and patients with bladder cancer [58]. Using a microarray platform probing for > 850 different human miRNAs in urinary EVs, the researchers identified 26 miRNAs that were significantly dysregulated in patients with high-grade bladder cancer (of these 26 miRNAs, 23 were down-regulated and the remaining 3 were up-regulated). Additionally, a candidate urinary EV associated miRNA, miR-375, was identified that appears to predict the presence of low-grade disease. Although EV research is still at a nascent phase, an important priority is to continue to characterise the cargoes contained in urinary EVs; defining the DNA, RNA and protein signatures of cancer-derived EVs has the potential to lead to the development of diagnostic tests as well as contributing insights into tumour biology in general.

Research into cancer-derived EVs, and exosomes in particular, is a rapidly expanding and exciting field. It is likely that as refinements in isolation techniques of EVs continue to develop, our understanding of the function of these particles as cell-to-cell communication vehicles will deepen. Furthermore, given that these are secreted particles and that secretion is elevated in cancer, research into EVs as potential urinary biomarkers for bladder cancer is a particularly attractive prospect. The rate-limiting aspect of EV research to date has largely been due to the labour intensive and technically challenging nature of EV and exosome isolation. However, recent advances, particularly in the field of microfluidics, appear set to improve the speed and ease of EV isolation. As uptake of these techniques become more accessible and widespread, it seems likely that research into urinary EVs is set assume greater prominence in the search for non-invasive biomarkers that can accurately diagnose bladder cancer and monitor treatment response.

## Conclusions

In this article, I have provided a broad overview of the state of research into urinary biomarkers as tools to aid the diagnosis of bladder cancer. Of course, urine cytology remains an important, impactful and cost-effective adjunct to cystoscopy, particularly in the detection of CIS and upper tract disease. In addition, I have drawn attention to several immunoassay-based techniques existing that rely on antibody-mediated detection of antigens associated with bladder cancer. Although, in general, these techniques display good specificity for the detection of cancer, they are somewhat hampered by their tendency to display low sensitivity for detection of low-grade tumours. As such, the clinical utility of these existing techniques is rather limited, and it seems unlikely that they will play a widespread role in bladder cancer diagnosis until more reliable, specific and universally expressed bladder cancer antigens are identified in urine.

Research into the detection of genetic material in the urine, such as DNA and RNA, derived from bladder cancer cells is of particular interest. Increasingly, DNA and RNA-based biomarker research is concerned with identifying genetic signatures that may not only allow us to detect disease but that may also provide an insight into the mutational landscape of a given tumour, which, as we move into precision medicine, may be employed to guide on-going therapies and predict responses to specific treatments.

Interest has developed in recent years in the role the extracellular vesicles (EVs) play in tumour biology as mediators of cell-cell signalling and drivers of cancer progression. Furthermore, techniques to isolate EVs have recently improved in sophistication such that EVs can now be extracted from bodily fluids, such as serum and urine, with relative ease. EVs offer a potentially attractive biomarker to detect primary disease and recurrence, whilst further research into understanding EV cargoes may well help characterise the biology of particular tumours to help predict the behaviour to provide prognostic, as well as diagnostic information.
